# Analysis of intellectual property strategies across different categories of digital therapeutics

**DOI:** 10.3389/fdgth.2026.1743156

**Published:** 2026-03-09

**Authors:** Tomoki Maeda, Hiroshi Suzuki, Chikako Saotome

**Affiliations:** 1 Department of Drug Discovery Medicine, Kyoto University Graduate School of Medicine, Kyoto, Japan; 2 Kyoto University Medical Science and Business Liaison Organization, Kyoto University, Kyoto, Japan

**Keywords:** digital health, digital therapeutics, intellectual property, patent, design right

## Abstract

Advances in digital technology and the coronavirus disease (COVID-19) pandemic have accelerated the digital transformation of healthcare. Digital therapeutics (DTx), which deliver evidence-based interventions through digital means to treat or prevent diseases, are expected to generate significant value in modern healthcare. Strategic intellectual property (IP) protection for DTx is essential to support development costs, including clinical trials, and to ensure sustainable innovation. This study analyzed patent and design right strategies across different categories of DTx. We examined 25 DTx products registered with the Digital Therapeutics Alliance in the United States and five U.S. Food and Drug Administration-approved augmented and virtual reality products as of April 2023, classifying them into three categories: app-based, app + device-based, and entertainment-based DTx. Patent data were collected from the Derwent Innovation Index, and design rights were identified using the Patent Public Search. A case study of one representative product from each category was conducted to contextualize these findings. The results revealed that half of app-based DTx lacked patent applications, often relying on platform technologies, while all app + device-based DTx had patents covering programs, biometric acquisition, and platform technology. Entertainment-based DTx exhibited the highest average number of patent applications, possibly due to their novelty and divergence from conventional treatments. Only five of the 30 products were protected by design rights, which appeared limited in scope and practical utility. Overall, distinct differences in patenting approaches were observed among DTx types, with design rights used sparingly. These findings suggest that optimal IP strategies vary by product architecture and that understanding such distinctions is essential for promoting innovation in digital therapeutics.

## 1 Introduction

Advances in digital technology and the coronavirus disease (COVID-19) pandemic have led to progress in the digital transformation of healthcare (1–3). Digital Therapeutics (DTx) are digital products that provide evidence-based interventions for treating, preventing, and managing diseases (4). They are expected to create unprecedented value by offering a promising approach for safely treating diseases, such as mental health disorders, that are difficult to manage with pharmaceuticals. Their economic impact is also significant, with the global DTx market projected to grow at a compound annual growth rate of 27.2% from 2024 to 2030 (5). Consequently, several pharmaceutical companies are interested in entering the DTx market (6).

Many DTx products incorporate machine-learned artificial intelligence (AI) to deliver Cognitive Behavioral Therapy (CBT) by presenting patients with recommendations that promote disease prevention and treatment (7, 8). Additionally, approval by regulatory authorities such as the United States (US) Food and Drug Administration (FDA) is required for the manufacture and sale of DTx; products for which similar counterparts are not on the market must prove efficacy and safety in clinical trials (9). Strategic intellectual property (IP) protection for DTx is crucial to cover development costs, including clinical trials. The IP rights applicable to DTx include patent rights that protect technological innovations invention, design rights that protect aesthetic appearance (shape, configuration, ornamentation, and patterns) industrial designs, copyrights that restrict the reproduction of programs, trademark rights that protect product names and marks, and trade secrets that protect useful business and technical information that is kept secret. Among these, patents, design rights and trademarks require applications to the patent offices in each country. DTx products are often protected by a combination of IP rights. However, the data protection systems recognized for pharmaceuticals do not pertain to DTx. Furthermore, since most DTx developers are start-ups, IP rights are crucial in forming alliances with large companies, such as pharmaceutical companies, to obtain research and development resources.

Because DTx products are a relatively new class of intervention modalities, few reports exist on IP strategies. Jeon et al. (10) analyzed patents applied to the US Patent Office to identify technological opportunities for DTx and recommended psychiatric diseases as a suitable area for development. In the DTx review article, dozens of US patents for AI-powered DTx were presented as examples (8). An analysis of 279 patents on AI-enabled precision psychiatry tools revealed that half of the patents related to treatment prediction recommendations mentioned digital therapy, with data sources including behavioral data collected through mobile devices (11). A study of DTx-related patents filed by Japanese companies revealed frequent use of “sensors”, “medical imaging”, and “central nervous system/ psychiatry”, suggesting that medical information technologies related to DTx were the focus area in Japan (12). However, little is known about the specific patent protection strategies for individual DTx products. In addition to patent rights, DTx can be protected by design rights for user interface (UI) design, but no specific research has been conducted.

Therefore, this study aimed to clarify the IP strategies for different DTx product categories. Since DTx products consist of some components, such as apps and devices, we hypothesized that IP strategies would vary depending on these component differences. Because it is impossible to investigate copyrights and trade secrets from public information, and trademark rights primarily protect product names, marks and icons with a limited impact on excluding technically similar products, we focused on two IP rights: patents and industrial designs.

The research questions were as follows:

1. Do DTx products utilize both patent and design rights? Do utilization ratios differ depending on the DTx components?
2. Are there differences in the subject of patent protection and design rights depending on DTx components?

Our study offers a novel contribution by providing a comprehensive analysis of both patent applications and design registrations across the entire spectrum of digital therapeutics (DTx), rather than limiting the scope to specific diseases or geographic regions. In addition, by examining differences in IP strategies according to product categories, this study generates insights with a high degree of generalizability, which have not been addressed in previous research.

## 2 Data and methods

We searched for DTx products as of April 11, 2023, in the US Digital Therapeutics Alliance's product library (13) and among US FDA-approved Augmented Reality (AR) and Virtual Reality (VR) products (14). Information on each DTx product, including the development company, target disease, and treatment method, was obtained from the FDA and other national regulatory authority databases and company websites.

We then classified the products into three categories based on their components: (1) app-based, (2) app + device-based, and (3) entertainment-based. App-based DTx was defined as those consisting of app only, while app + device-based DTx included a device in addition to the app. Entertainment-based DTx was defined as products that included game apps or VR/AR devices.

We searched for patent applications for each DTx product using the Derwent Innovations Index (Clarivate), entering the name of the company that developed DTx as the applicant or assignee (as of July 14, 2023). The Derwent Innovations Index contains full-text patent data from over 70 countries, providing comprehensive global coverage for our analysis. For design rights, we searched the US Patent and Trademark Office's Patent Public Search by the company's name as the applicant (as of June 22, 2023). From the patent applications filed by each company, we extracted product-related patents that protect the DTx product itself, and platform technology patents that protect the underlying technology of DTx.

Regarding product-related patents, we compared the three elements—target disease, component of DTx, and treatment method—described in the claims that specify the invention in the patent specification with the relevant DTx. We identified patent applications with the same items as the product-related patents. However, the application was excluded if only one of the three elements matched the DTx product and the others differed. For example, if a DTx product targeted attention deficit hyperactivity disorder and included a game app as a component, but the patent claim described diabetes as the target disease while the component was a game app, the patent application was excluded from product-related patents. For platform technology patents, we extracted applications classified under G16H (healthcare informatics) in the International Patent Code (IPC) or those claiming DTx platform technology inventions. The IPC is a hierarchical system of language-independent symbols used for patent classification (15). IPC divides technology into eight sections with approximately 70,000 subdivisions. The patents were independently evaluated by two authors. In cases of disagreement regarding inclusion or exclusion, the final decision was reached through consensus between the two reviewers. We extracted design rights related to DTx products, specifically those described in claims as UI or device designs used in the product. A list of all patent applications and designs associated with DTx products is provided as Supplementary Data.

Next, we classified the subject of patent and design protection. Patent applications were categorized into four groups: (I) program-related, (II) therapeutic systems, (III) acquisition of biological information, and (IV) platform technology (Table 1). Platform technologies were further divided into four subcategories: IV-1) common treatment-related systems, IV-2) medical data management/transmission and reception, IV-3) imaging, and IV-4) other. If a single patent application fell into multiple categories, each category was counted as a single. For example, if a patent was classified as a program-related or therapeutic system, it was counted once in each category, and the percentage of patents in each category was calculated. Design rights were classified into (I) UI design and (II) device design. The two authors evaluated the relevance and subject of the IPs independently and in duplicate. In cases of differing opinions, a consensus was reached between them.

**Table 1:** Categories of patent applications.

Group	Category	Definition
I	Program-related	The treatment program or its method that include the target disease of the DTx product.
II	Therapeutic Systems	The therapeutic system that consists of apps, devices, and servers, and includes the target disease of the the DTx product.
III	Acquisition of Biological Information	Devices or methods used to acquire biometric information.
IV	Platform Technology	The technology that is not limited to specific DTx products but can be used across multiple DTx products.
IV-1	Common Treatment-related Systems	Treatment systems consist of apps, devices, and servers where the target diseases are not limited.
IV-2	Medical Data Management/Transmission and Reception	Systems or methods related to managing, transmitting, and receiving medical data.
IV-3	Imaging	Technologies related to image display, such as UI and GUI (graphical user interface).
IV-4	Others	Patents not falling under the above categories include therapeutic app distribution systems, therapeutic devices, systems for presenting medical services to providers, and software quality assurance.

## 3 Results

### 3.1 DTx products

**Figure 1:**
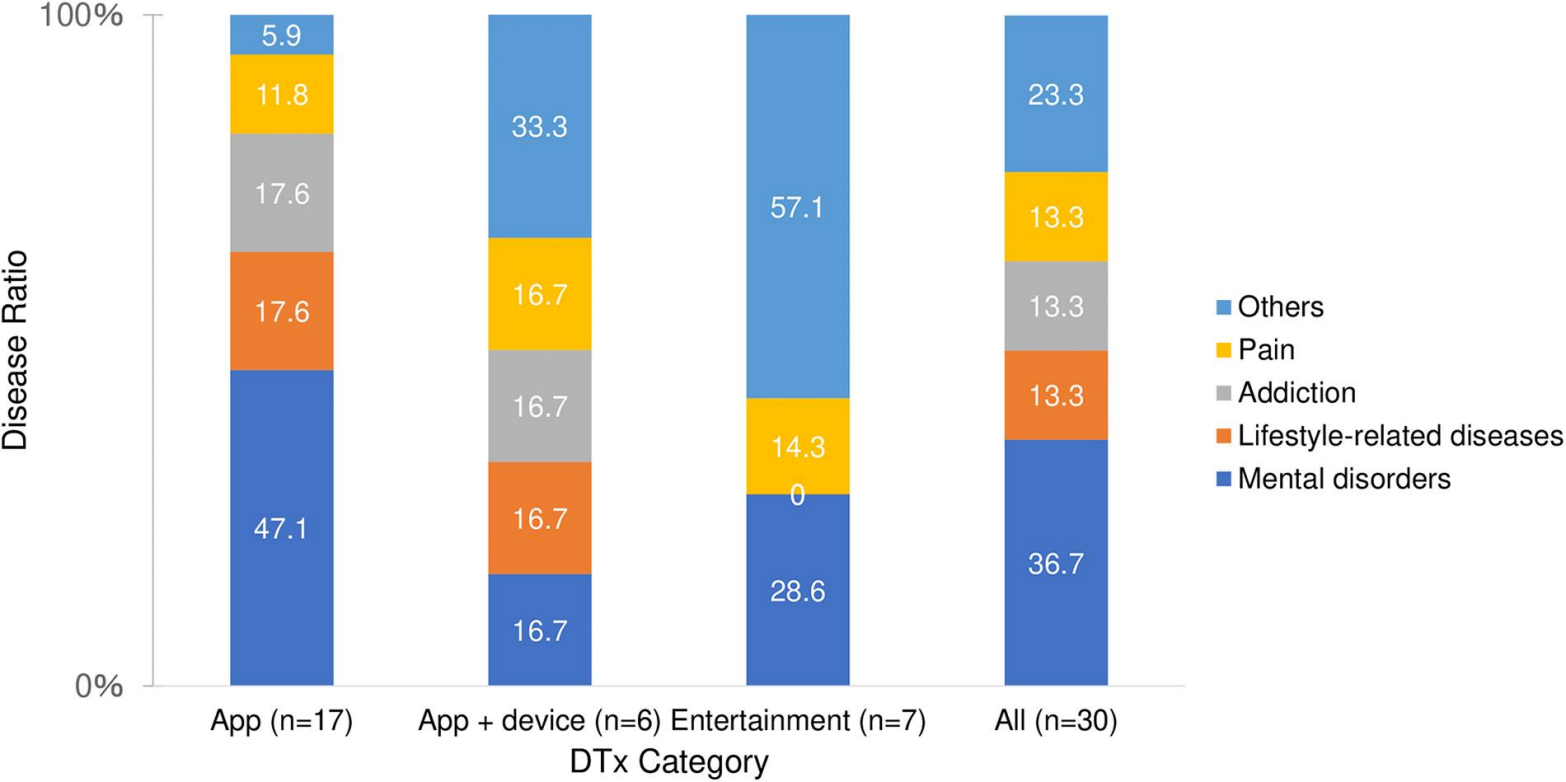
Target diseases of all and each product category.

As of April 11, 2023, there were 30 DTx products: 17 app-based, six app + device-based, and seven entertainment-based products. Figure 1 shows the percentage distribution of the target diseases across all products and product categories. Mental disorders, lifestyle-related diseases, and addiction account for over 60% of target diseases across all DTx products. Approximately half of the app-based DTx focused on mental health. Regarding therapeutic interventions, 16 products (53.3%) used CBT, 14 app-based products (82.4% in the category), and two app + device-based products (33.3% in the category). No entertainment products utilize CBT.

### 3.2 Patent application

First, we examined whether patent applications were filed and the number of patent applications per product. Of the 30 DTx products, 21 (70.0%) filed product or platform technology patent applications. While all app + device-based and six of seven (85.7%) entertainment-based DTx had a patent application, nine app-based products (52.9%) had a patent application. All app-based DTx without patent applications used CBT. The average and median numbers of patent applications for the 21 DTx with patent filings were 9.5 and 5.0, respectively. The average numbers of applications for app-based, app + device-based, and entertainment products were 10.1, 7.8, and 10.3, respectively; the median numbers of applications were 5.0, 5.0, and 7.0, respectively. Among the nine app-based products with patent applications, only two (BlueStar with 46 and CureApp HT with 16) had more applications than average.

**Figure 2:**
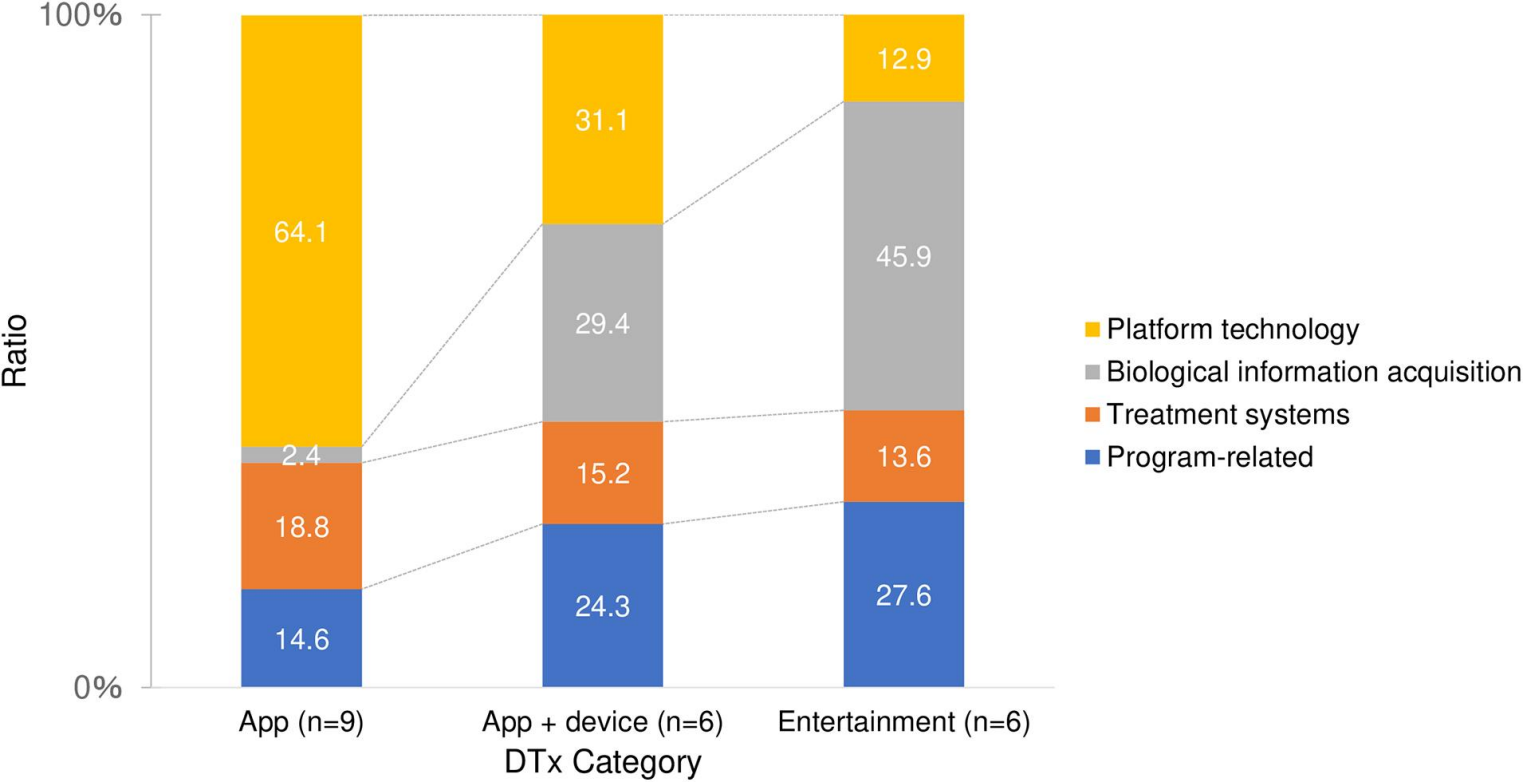
Percentage of patent subjects for each product category.

**Figure 3:**
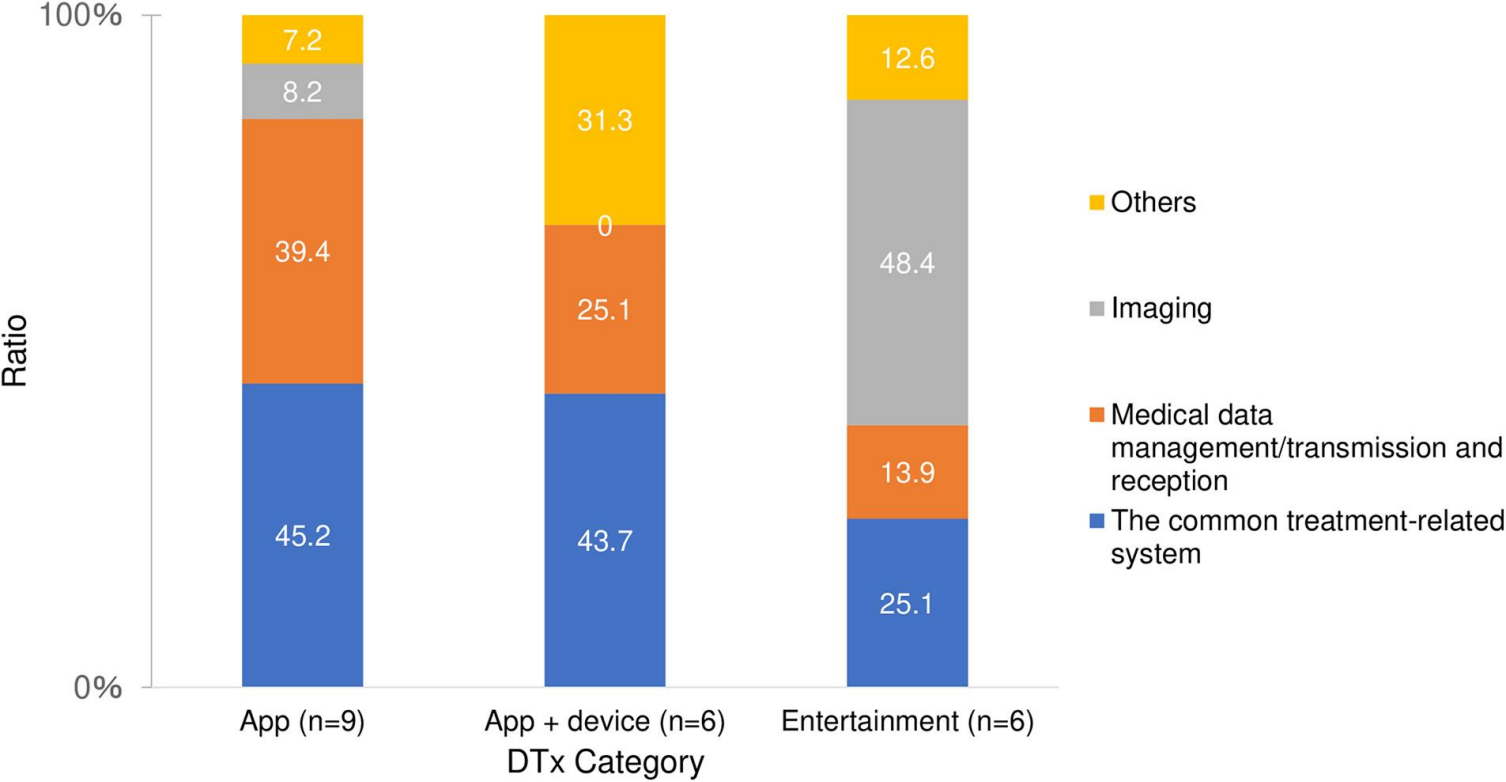
Percentage of platform patent subcategories for each product category.

Analysis of the patent content for the 21 products with patent applications revealed that the percentages of program-related, therapeutic systems, acquisition of biological information, and platform technology patents were 21.4%, 16.6%, 21.8%, and 40.3%, respectively. Figure 2 shows the distribution of patent subjects for each product category. Platform technology accounted for over 60% of patents for app-based products. Although platform technology (31.1%) had the highest proportion in the app + device-based category, the proportion of biological information acquisition (29.4%) was almost the same; program-related patents accounted for a quarter of the total (24.3%). For entertainment-based products, the acquisition of biological information accounted for nearly 50%. An analysis of the platform technology subcategories for the 21 products revealed that the percentages of common treatment-related systems, medical data management/transmission and reception, imaging, and other patents were 42.4%, 32.3%, 10.6%, and 14.7%, respectively. Figure 3 shows the percentage distribution of the platform technology subcategories by product category. For app-type products, common treatment-related systems accounted for nearly 50%, while medical data management/transmission and reception accounted for 40%. For app + device-based DTx, common treatment-related systems accounted for over 40%, whereas in the entertainment-based, imaging patents accounted for nearly 50%.

### 3.3 Design rights

Of the 30 products, only five (16.7%) had design applications. By product categories, the distribution was as follows: app-based (two products, 11.8% in the category), app + device-based (one product, 16.7% in the category), and entertainment-based (two products, 28.6% in the category). The average number of design applications for the five products was 2.2. By product type, the averages were 2.5 for app-based, 3.0 for app + device-based, and 1.5 for entertainment-based DTx. Regarding the protection subject, UI design was covered for two app-based products and one entertainment product, while device design was protected for the app + device-based and one entertainment product.

### 3.4 Case studies

**Figure 4:**
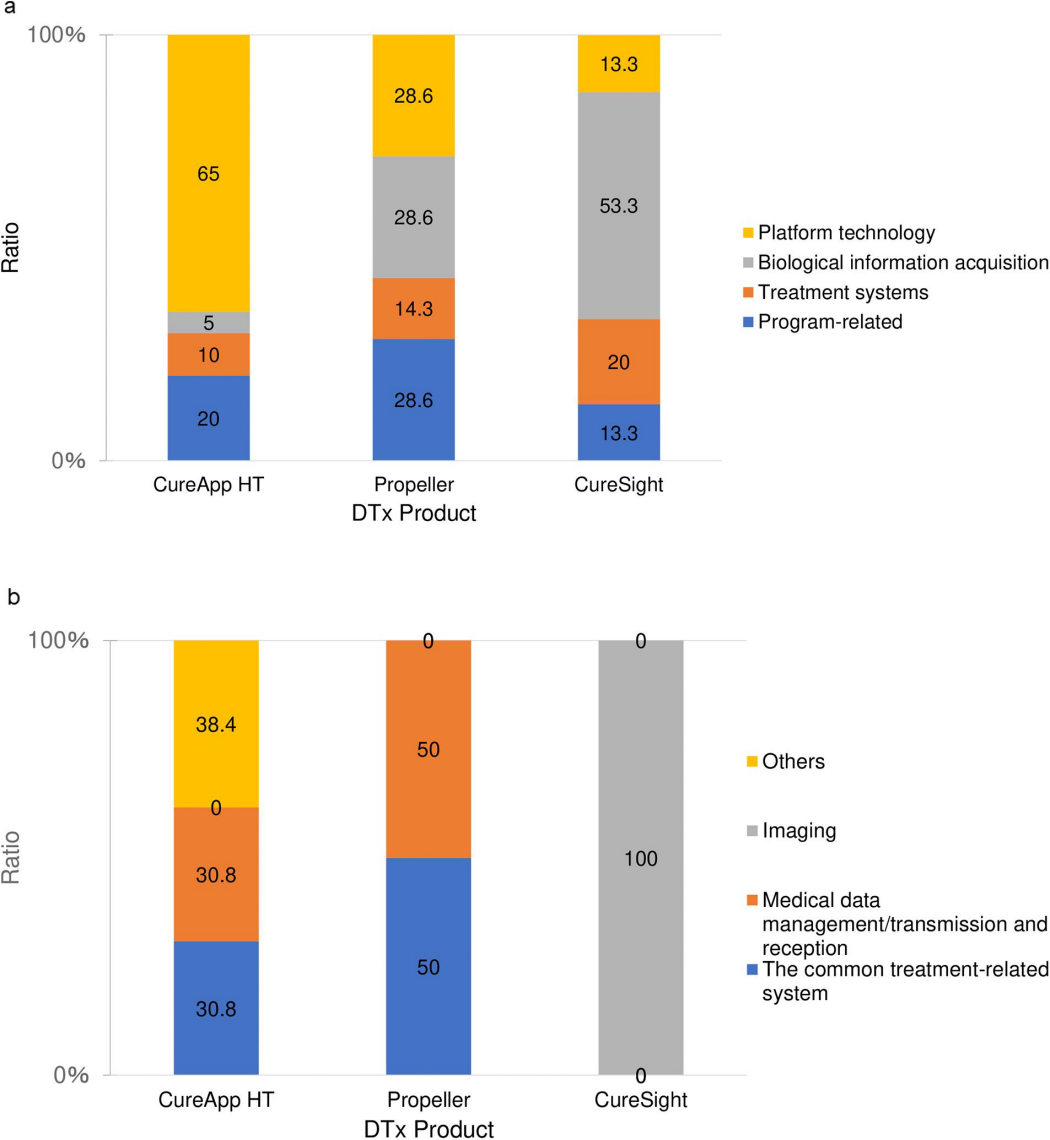
Percentage of patent subjects for CureApp HT, propeller, and cureSight. **(A)** Percentages of Category (I)–(IV). **(B)** Percentages of Platform technology (IV-1)–(IV-4).

To facilitate a comprehensive analysis of IP strategies, we conducted case studies on a representative product from each product category: CureApp HT® from the app-based category, Propeller® from the app + device-based category, and CureSightTM from entertainment products. These products were carefully selected to ensure that their patent subject ratios were representative of category averages (Figure 4).

#### 3.4.1 App-based DTx: CureApp HT®

CureApp HT® is an app-based DTx for hypertension treatment developed by the Japanese company CureApp Corp. and approved by the Japanese Ministry of Health, Labor, and Welfare in April 2022. This product requests patients to enter information about their conditions, such as blood pressure and diet, into an application. It then uses this information to present knowledge about hypertension that is assumed to be lacking, appropriate behavioral goals, and reminders for treatment behavior (16, 17). CureApp Corp. filed 16 patent applications for this product, and the composition of the subjects of these patent applications showed that platform technology accounted for more than 60% (Figure 4a, left). Within the platform technology, the common treatment-related system and medical data management/transmission and reception each accounted for approximately 30% (Figure 4b, left).

CureApp Corp. filed four patent applications (18–21) for common treatment-related systems related to CureApp HT®. For example, KOHADA's invention (21) claims to a program, method, and information processing apparatus that performs CBT. The CBT process described in this patent application is as follows: under the circumstances where an information processing device, including a feedback acquisition unit, a difficulty level setting unit, and an action goal presentation unit, is connected to each patient terminal via a network (i) the feedback acquisition unit acquires questionnaire results concerning diet, exercise, weight, medical history, etc.; (ii) the difficulty level setting section determines the difficulty level of implementing the pre-defined behavioral goals for the patient based on the questionnaire results, using a learning model that has been trained to recognize the correspondence between the questionnaire items and the difficulty level of each behavioral goal in advance; and (iii) the behavioral goal presentation section provides the behavioral goals to the patient terminals in order of decreasing difficulty level.

They also filed four medical data management patent applications (22–25) related to CureApp HT®. The most recent application (25) pertains to a patient information disclosure system and process for third parties, especially peer supporters such as family members who assist in patient treatment. The disclosure system consists of terminals used by patients, medical professionals, peer supporters, and a server that can communicate with these terminals. The disclosure process is as follows: (i) the server accepts requests for the disclosure of patient information stored on the peer supporter's terminal; (ii) the server generates authorization information, such as a one-time password, and sends it to the peer supporter's terminal; and (iii) the peer supporter sends the received authorization information to the server and then receives the patient information.

#### 3.4.2 App + device-based DTx: propeller®

Propeller® is a DTx used for treating chronic obstructive pulmonary disease. It consists of a smartphone application and a device with a medication inhaler attachment that can obtain information on medication times and locations for asthma (https://dtxalliance.org/products/propeller/). The Propeller® analyzes the time and location of medication intake using a medication inhaler attachment and assesses the environmental factors that trigger asthma. It also provides warnings about environmental factors, educational content about the disease, and medication reminders for patients. Propeller Health, the company that developed Propeller, filed three patent applications for this product, and the composition of these patent applications was almost 30% for the program-related patents and for the acquisition of biological information (Figure 4a, middle).

The patent application related to the program pertains to a program, method, and system designed for avoiding the risk of respiratory disease (26). The process of avoiding the risk of respiratory diseases described in this patent application involves (i) receiving the user's location from the patient's computer; (ii) accessing the air quality dataset to obtain the air quality at the user's location; (iii) retrieving environmental data, including land use and vegetation, for the user's location; (iv) accessing the correlation model between air quality, environmental data, and disease occurrence to obtain the disease occurrence rate; and (v) determining the recommended actions for the patient and sending them to the patient's device. Thus, the notable characteristic of this invention is a prediction model for asthma attacks in patients that incorporates geographical information.

The patent applications for acquiring biological information relate to methods and devices for detecting drug inhalation (27). The process of detecting drug inhalation includes a wireless transceiver connected to the patient terminal, an acceleration sensor, an infrared sensor, a microphone, and a processor. The drug inhaler attachment, which is connected to the patient terminal, has an acceleration sensor activated only when the device is in low-power mode, and an acceleration sensor, infrared sensor, and microphone are activated when the device is in power-on mode. When the patient terminal is connected to the server, the inhalation-detecting process occurs as follows: (i) the acceleration sensor detects movement of the device and switches the inhaler attachment from low-power mode to power-on mode, (ii) the infrared sensor detects the opening of the inhaler, (iii) the acceleration sensor detects inhalation of the medicine, (iv) the microphone records the inhalation sound, (v) the wireless transceiver transmits the inhalation sound to the patient terminal, (vi) the patient terminal transmits it to the server, and (vii) the server compares it with the patient's previously recorded inhalation sound to determine whether was inhaled. This patent includes sensors that record medication inhalation as part of the patient's vital information and mechanical innovations for power saving.

#### 3.4.3 Entertainment-based DTx: CureSight™

In 2022, CureSight™ received US FDA clearance (K221375) for treating amblyopia in pediatric patients aged four to nine (https://nova-sight.com/curesight-amblyopia-treatment/). CureSight™ includes AR glasses that track eye movements and blur the center of the field of vision. One of the causes of amblyopia is the difference in visual acuity between the left and right eyes from birth. If the stronger eye is used exclusively during the critical period of visual acuity development in childhood, the weaker eye may not develop properly. The conventional treatment for this condition uses an eye patch on the stronger eye during childhood to encourage the use of the weaker eye more frequently.

CureSight™ treats patients by wearing AR glasses while they watch their favorite videos. The device blurs the center of the visual field of the stronger eye, forcing the use of the weaker eye. NovaSight, the company that developed CureSight™, filed nine patent applications related to this product, with more than half of its content pertaining to acquiring biological information (Figure 4a, right). A patent application for obtaining biological information describes a system and method for measuring ocular motility in patients (28). The method for determining the ocular motility parameters involves (i) displaying at least one target for at least one eye, (ii) receiving image data indicating the status of at least one eye from the camera unit, (iii) controlling the blocking unit to block or unblock at least one target in the visual field of at least one eye of the patient, (iv) detecting changes in the condition of at least one eye, and (v) moving the target in at least one eye. NovaSight filed a patent for a device and process that obtained biological information and performed a visual field obstruction process (29). The visual field obstruction process in this patent application involves the following steps: (i) the eye tracker acquires the direction of gaze and the size of the pupil, (ii) the controller adjusts the optical elements on the lens based on these data, and (iii) the center of the visual field of the better eye is blurred. Thus, patent applications were filed mainly for the devices and methods used in CureSight to detect eye movement.

## 4 Discussion

This study aims to clarify whether IP strategies differ depending on the DTx product category. It also discusses the differences in patent application strategies and design rights for each product category. Finally, it considers the IP strategies for products without patent applications or design rights.

### 4.1 Patent applications

First, for app-based DTx products, almost half (8/17, 47.1%) had no patent applications. Among app-based DTx, 80% of the products used CBT as the treatment method, and all eight products without patent applications used CBT. After the US Supreme Court's ruling in *Alice Corp. v. CLS Bank International* (30)., patent claims that simply perform abstract ideas through generic computer implementation are deemed ineligible for patent protection (31). Because CBT is already performed by physicians, establishing patentability for inventions related to app-based implementations of CBT can be challenging, which may reduce the percentage of patent applications. More than 60% of the patent applications for app-based DTx were for platform technology. Approximately 40% of the platform technology applications were for common treatment-related systems and medical data management/transmission and reception, respectively. Regarding the common treatment-related systems, as seen in CureApp's patent application case study, some applications were not for a CBT treatment program/system for a specific disease but rather for an infrastructure system that provides CBT without specifying the disease. This is because they attempted to make the system patentable by incorporating a learning model trained to associate questionnaire items with the difficulty level of each behavioral goal. By not specifying the disease, the patentability of such inventions differs from that of conventional CBT, which is performed by physicians for specific diseases.

Regarding medical data management/transmission and reception, CureApp's patent application is an information disclosure system that involves peer supporters who are not patients or medical professionals. The inclusion of peer supporters may be a growing need for information disclosure technologies for individuals other than patients and physicians because DTx can be used outside hospital settings. App-based products showed the largest difference between the average (10.1) and median (5.0) number of applications among the three product types because of the higher number of patent applications from BlueStar and CureApp HT® (BlueStar®: 46, CureApp HT®: 16). These findings suggest that both companies aimed to obtain comprehensive IP rights for standard DTx technologies.

In contrast to the app-based products, all app + device-based products have patent applications. Most app + device-based products use treatment methods other than CBT. The most common type of invention was platform technology, and a common treatment-related system was the most prevalent, similar to app-based DTx. However, compared to app-based DTx, app + device-based products showed a substantial increase in the proportion of patents related to biological information acquisition. In a case study, Propeller Health applied for a patent for a method and a device for detecting drug inhalation. They also filed a patent for a program that calculated the incidence of disease in patients based on environmental data and provided recommendations to patients. They utilized the information obtained from these devices to create learning models. These findings suggest that in the app + device-based DTx, patents are more likely to focus on programs related to new treatment methods other than CBT and devices for acquiring biological information.

All entertainment-based DTx products except one had patent applications; their average number was the highest among the three product categories. Approximately half of patent applications were related to the acquisition of biological information. The fact that no entertainment-based DTx used CBT suggests that the treatment methods for these products differ significantly from traditional methods, leading to more opportunities for innovation during product development. For example, the case study of NovaSight highlights a DTx product that uses AR technology to block vision while tracking eye movements, which is significantly different from the conventional method of treating amblyopia using eye patches. Approximately 50% of patent applications related to NovaSight™ focus on biological information acquisition. This focus is likely due to the necessity of acquiring biological information to optimize game-based treatments and integrate AR and VR functionalities. NovaSight™ tracks eye movements and pupil size, whereas VR products require acquiring biometric information to reflect body movements in virtual environments.

To summarize, the characteristics of the patent applications for different types of DTx products were as follows:

1. App-based DTx products: The overall number of patent applications was relatively low, except for a few companies that applied for many patents on the DTx platform technologies. One reason is that most app-based DTx use CBT as a treatment method, and it is difficult to differentiate them from conventional methods.
2. App + device-based DTx products: Patent applications focus more on devices for acquiring biological information than app-based products. Because CBT is not used, it is easier to develop new treatment programs.
3. Entertainment-based DTx products: As these involve novel treatment methods, many patent applications have been filed, primarily related to technologies for acquiring the biological information necessary for AR and VR.

### 4.2 Design rights

Only five of 30 products (16.7%) had design rights, and the number of applications across all product categories was low. The number of design applications worldwide is approximately one-third of the number of patent applications, indicating that they are not widely used as patents (32). The reasons for this include the narrow scope of rights related to the cost of applying for design rights and the preference for other IP protections, such as trademark rights (33). Although there were applications for UI and devices in DTx, they had a lower priority than the patents. In DTx products, one reason design rights are used less extensively than patents is the characteristics of the UI and device design. Although UI and device design play important roles in enhancing patient engagement with the application and improving usability and convenience, their direct influence on therapeutic efficacy is relatively limited. In many cases, alternative designs that deliver comparable therapeutic outcomes can be devised. Consequently, the ability of design rights to deter competitors from adopting design-around strategies is relatively weak, and design rights do not function effectively as strong barriers to market entry. In contrast, patents can protect the fundamental mechanisms that generate therapeutic effects, such as system architectures, algorithms, and digital treatment protocols, at a higher level of abstraction. Because these core mechanisms are more difficult for competitors to circumvent, patents are highly effective in safeguarding the essential value of DTx businesses. Therefore, companies generally prioritize patents over design rights in this domain.

Nevertheless, design rights still hold potential for more strategic use in the DTx industry. To compensate for the inherent ease of design-around, firms could, for example, introduce multiple DTx products with similar UI or design at an early stage, thereby establishing the design as a de-facto standard within the industry. Such a “dominant design” strategy could help deter imitation and leverage the advantages of design rights in brand recognition and user experience, ultimately contributing to a stronger competitive position in the DTx market.

### 4.3 DTx with no patents or designs

Finally, we discuss the DTx products for which no patents or design applications have been filed. Eight app-based products, accounting for approximately half of the app-based DTx, and one entertainment-based DTx product had no patents or design applications. Notably, the same company marketed five of the eight app-based DTx without patents or design applications. Given its extensive product portfolio, which includes five DTx products, the company appears to have a more rapid DTx development cycle than its competitors, many offering only one or two products. This finding suggests that the company prioritizes market penetration and first-mover advantages over IP protection, as evidenced by its rapid product development without significant investment in IP. Many DTx use portals that allow medical professionals to view patient information. As it can be complicated for doctors to manage multiple portals, companies can gain a competitive advantage by launching portals early and familiarizing doctors with their systems. Additionally, for diseases with long treatment periods, patient data accumulated by DTx can have therapeutic value; therefore, an early market launch may also be beneficial from the perspective of accumulation. In contrast, a company that develops entertainment-based products without patents or design rights licensed its technology to Akili Interactive Labs for 37.5 million US dollars (34). This indicates that the company possesses valuable technologies that warrant a high licensing price without patents or design applications. Therefore, it is suggested that companies seek to protect their products using trade secrets and program copyrights rather than disclosing the details of their programs externally through patent applications.

In summary, companies employ various strategies to secure profits from their products by securing first-mover advantages and protecting other forms of IP, such as trade secrets, by utilizing alternative strategies beyond patents and design rights.

## 5 Limitations

Since this study investigated the status of applications, not all applications may necessarily be granted patents. Their significance in product protection should be assessed after examination by the patent office. Moreover, analyzing the countries where these rights are obtained is necessary.

Regarding the business strategies of products for which no patents or design applications were filed, we considered that these companies were attempting to build a competitive advantage by securing a first-mover advantage or protecting their products through trade secrets or program copyrights. However, these assumptions should be examined further through interviews with relevant companies and other verification methods.

## 6 Conclusions

This study classifies DTx products into three categories based on their constituent elements: app-based, app + device-based, and entertainment–and investigates the differences in their corresponding IP strategies. The app-based product category demonstrates a small number of patent applications, primarily because their underlying technology (such as AI algorithms, computer programs, mathematical methods, or data processing methods) constitutes a patent-ineligible subject matter under the current legislative framework. However, a few companies that applied for patents in this category predominantly intended to protect platform-based DTx technologies by providing technical solutions to specific healthcare problems. In contrast, app + device- and entertainment-based DTx products show near-universal patent coverage, predominantly protecting biological sensing technologies and hardware-integrated innovations satisfying the patentability criteria of novelty and industrial utility. Across all the three product categories, design rights remain underutilized, as patent protection offers broader exclusivity and return on investment opportunities over technical innovations, and also because it is a preferred defensive strategy over design rights.

## Data Availability

The raw data supporting the conclusions of this article will be made available by the authors, without undue reservation.
